# HACE1 expression in heart failure patients might promote mitochondrial oxidative stress and ferroptosis by targeting NRF2

**DOI:** 10.18632/aging.205272

**Published:** 2023-12-06

**Authors:** Peiyi Yin, Yongbin Wu, Xiang Long, Shuqiang Zhu, Shiwei Chen, Feng Lu, Kun Lin, Jianjun Xu

**Affiliations:** 1 Department of Cardiothoracic Surgery, The Second Affiliated Hospital of Nanchang University, Nanchang 330006, China

**Keywords:** heart failure, oxidative stress, ferroptosis

## Abstract

Background: Heart failure is a prevalent and life-threatening medical condition characterized by abnormal atrial electrical activity, contributing to a higher risk of ischemic stroke. Atrial remodelling, driven by oxidative stress and structural changes, plays a central role in heart failure progression. Recent studies suggest that HACE1, a regulatory gene, may be involved in cardiac protection against heart failure.

Methods: Clinical data analysis involved heart failure patients, while an animal model utilized C57BL/6J mice. RT-PCR, microarray analysis, histological examination, ELISA, and Western blot assays were employed to assess gene and protein expression, oxidative stress, and cardiac function. Cell transfection and culture of mouse atrial fibroblasts were performed for *in-vitro* experiments.

Results: HACE1 expression was reduced in heart failure patients and correlated negatively with collagen levels. In mouse models, HACE1 up-regulation reduced oxidative stress, mitigated fibrosis, and improved cardiac function. Conversely, HACE1 knockdown exacerbated oxidative stress, fibrosis, and cardiac dysfunction. HACE1 also protected against ferroptosis and mitochondrial damage. NRF2, a transcription factor implicated in oxidative stress, was identified as a target of HACE1, with HACE1 promoting NRF2 activity through ubiquitination.

Conclusions: HACE1 emerges as a potential therapeutic target and diagnostic marker for heart failure. It regulates oxidative stress, mitigates cardiac fibrosis, and protects against ferroptosis and mitochondrial damage. The study reveals that HACE1 achieves these effects, at least in part, through NRF2 activation via ubiquitination, offering insights into novel mechanisms for heart failure pathogenesis and potential interventions.

## INTRODUCTION

Supraventricular tachyarrhythmia, a disorder characterized by rapid disruptions in atrial electrical activities, is a critical component of heart failure [1]. In China, heart failure prevalence stands at approximately 0.77%, with a notable increase correlating with age, particularly among individuals over 80 years old (7.5%) [2]. Heart failure represents a primary and independent risk factor and a significant contributor to the occurrence of ischemic stroke [3]. Patients with heart failure face a significantly higher risk, up to 5-7 times, of suffering a stroke compared to those without this condition [3]. A pivotal mechanism behind heart failure recurrence is atrial remodeling, encompassing autonomic, structural, and electrical alterations [4]. Atrial dysfunction, stemming from abnormal conduction and systolic function loss, contributes to heart failure development [5], primarily driven by structural remodeling processes [6].

Oxidative stress, mediated by reactive oxygen species (ROS), exacerbates atrial structural and electrical remodeling and plays a detrimental role in the onset and progression of heart failure. Elevated ROS levels lead to DNA, lipid, and myocardial protein damage, as well as the activation of inflammatory cell cytokine production, tissue damage, heightened inflammatory responses, and atomic fibrosis [7, 8]. In response to hemodynamic changes sensed in cardiac tissues, HACE1 acts to protect against heart failure through cardiac protection mechanisms [9]. Heart failure patients exhibit elevated serum HACE1 levels, which help control protein degradation and safeguard the heart from hemodynamic stress [10]. HACE1-mediated ubiquitinated proteins interact with ankyrin domain proteins, facilitating selective autophagy of heart failure-related cells, underscoring its pivotal role in cardiac protection [11].

Mice with defective HACE1 expression display abnormal myocardial hypertrophy, left ventricular dysfunction, and the accumulation of autophagy-related proteins LC3 and p62, indicating disrupted autophagy processes that exacerbate heart failure and increase mortality rates. Given this context, HACE1 emerges as a promising candidate for both heart disease diagnosis and treatment [12]. Understanding the significance of heart failure, circulatory embolism, and ischemic stroke in clinical practice provides valuable context for appreciating the relevance of this study.

## MATERIALS AND METHODS

### Clinical data analysis

This study was approved by the Ethics Committee of Second Affiliated Hospital of Nanchang University, Nanchang, China (Approval No. AHNU/2019/A213). After getting approval from the ethical committee the current study was conducted and all participants provided written informed consent prior to enrolment in the study. This research was conducted ethically in accordance with the World Medical Association Declaration of Helsinki. Electrocardiogram results were obtained for those patients (n=29) who were diagnosed with heart failure. Patients with ddiagnosis of Heart Failure based on clinical assessment, including symptoms, medical history, and diagnostic tests such as echocardiography and B-type natriuretic peptide (BNP) levels, 40-70 years old must have undergone ECG testing to confirm the presence of supraventricular tachyarrhythmia or relevant atrial electrical abnormalities, were enrolled in the study. Patients with below or above the specified age range with or without severe comorbid conditions, such as end-stage renal disease, advanced cancer, or other life-limiting illnesses, were excluded. Based on these results, the patients were recruited from the hospital for the current study.

### Animal experiment

Protocols for animal experiments were approved by the Animal Experimental Ethics Committee of the Nanchang University, Nanchang, China (Approval No. AHNU/2019/A215) in compliance with the National Institutes of Health guidelines for the care and use of laboratory animals. For this study, the animals (C57BL/6J mice) of 5-6 weeks old were divided into two groups in a randomized manner with each group containing 8 mice. The groups were sham and model or model and model+si-HACE1. The sham group mice were injected with normal saline intraperitoneally. On the other hand, the model group mice were intraperitoneally injected with Ang II (1.5 μg/g/ day; Sigma-Aldrich, USA). Then, the mice were sacrificed after 4 weeks under anesthetic conditions. Afterwards, the model+UBQLN1 group mice were injected with Ang II (1.5 μg/g/ day; Sigma-Aldrich, USA) and UBQLN1 RNA vectors (4 ×10^7^ TU/mice/week) intraperitoneally. Again, the mice were sacrificed under anesthesia after 4 weeks. The chemically-synthesized lentiviral vectors containing si-HACE1 RNA and a negative control, was designed and procured from Hanyin Biotechnology Limited Company (Shanghai, China). The diameter, ejection fraction, fractional shortening and the stroke volume of the left ventricles were determined using Nillar pressure-volume system (MPVS-400).

### RT-PCR assay and microarray analysis

In brief, the extraction of the total RNA was performed using RIzol reagent (Invitrogen, Thermo Fisher Scientific, Inc., Waltham, MA, USA) either from heart tissue or cell samples of the mice or from human serum samples. Then, the total RNA was made to undergo reverse transcription into cDNAs with the help of Reverse Transcription System Kit (Takara, Dalian, China). qRT-PCR was used to amplify the cDNAs in which TB Green™ Premix Ex Taq™ II (Takara, Dalian, China) was utilized on a StepO-nePlus Real-Time PCR System. Based on 2 -ΔΔCt methods, the fold changes of the mRNA level were determined.

Out of the serum samples, the extraction of the total RNA was performed. Afterwards, the RNA quantification was performed with the help of NanoDrop 1000. Every sample’s total RNA was utilized to perform the reverse transcription process in which Invitrogen SuperScript double stranded cDNA synthesis kit was utilized. NimbleGen one-color DNA labeling kit was utilized to execute the double-stranded cDNA after which array hybridization was performed with the NimbleGen hybridization system. Then, the sample was washed using NimbleGen wash buffer kit. For the purpose of scanning, the researchers used Axon GenePix 4000B microarray scanner (Molecular Devices, , San Jose, CA, USA).

### Histological examination and enzyme-linked immunosorbent assay (ELISA)

After fixing the tissue in 4% paraformaldehyde for about 24 hours, the sample was cut into slices measuring 5 μM thick in transverse plane. Then, the sectioning of the paraffin-embedded tissues was accomplished at 4-μm and the sections were stained using H&E staining or else Masson staining. In order to analyze the sections, the authors used Olympus BH-2 light microscope (Olympus Corporation). ELISA kit’s instructions (manufactured by Beyotime) were followed to determine the levels of ROS produced, GSH, MDA, GSH-px and SOD levels.

### Cell transfection and culture of adult mouse atrial fibroblasts

For the purpose of down-regulating the expression of HACE1, the authors introduced thesi-HACE1 virus (sc-41670, Santa Cruz Biotechnology, USA) into mice. As per the methodology in literature [13], the atrial fibroblasts of the adult mice were isolated. From the animals considered for study i.e., C57BL/6J mice (5–6 weeks old measuring 18-20 g) were sacrificed, hearts excised and submerged in cold PBS. Then, a tissue chopper was used to chop the atria into pieces after which it was washed with cold PBS. Then, collagenase II (1 mg/mL, Worthington Biochemical, USA) was used for 30 minutes to dissociate the cells at a temperature of 37° C. Then, the collection of the isolated cells was performed followed by its centrifugation at 1500 rpm for 5 min. Again, the solution was suspended in DMEM medium (supplemented with 10% FBS) for about 2 hours at a temperature of 37° C under 5% CO_2_ incubator. After the removal of myocyte-rich medium, the cells were inoculated for about 3-4 days prior to passage. Lipofectamine™ 2000 (Invitrogen, USA) was used to transfect the cells with their corresponding constructs as per the instructions from the manufacturer. Then, the cells were treated using 1 μM Ang II (Sigma-Aldrich, St. Louis, MO, USA) after four 4 of transfection. These cells were then utilized to perform the rest of the experiments after 48 hours of incubation.

### Western blot assay

With the help of protein lysis buffer (Keygen Biotech, Nanjing, China), the extraction of the proteins was conducted from the heart tissues or otherwise the cell samples. BCA reagent kit was utilized to determine the quantity of the protein present in the samples. Then, the protein samples (Lysates) were separated using 10% SDS-PAGE after which it was shifted to polyvinylidene difluoride (PVDF) membranes (Millipore Corp. Billerica, MA, USA). At 4° C overnight, the incubation of the PVDF was performed by HACE1, NRF2, GPX4 and β-actin. Then, for about 2 hours, the horseradish peroxidase-conjugated secondary antibody (Beyotime, 1:5000) was used for the incubation of PVDF. Then, the signal was analyzed by leveraging the chemiluminescence system (Amersham Pharmacia, UK).

### Immunofluorescence

After fixing the cells in 4% paraformaldehyde for about 15 mins, the permeabilization of the cells was conducted using 0.1% Triton X-100 in PBS for about 15 min at room temperature. Then, 5% BSA was used to block the cells for about 30 minutes at 37° C. Then, the cells were made to undergo treatment with primary antibodies such as anti- HACE1 (1:100) and anti-Nrf2 (1:100) at 4° C overnight. After the incubation of the cells with Cy3-conjugated goat anti-rabbit or goat anti-mouse IgG DyLight 488-conjugated secondary antibodies (1:500), for about one hour at a temperature of 37° C, DAPI was used to stain the nuclei. Fluorescent confocal microscopy (Nikon, Tokyo, Japan) was used to visualize the cells.

### Data and statistical analysis

For the current study’s statistical analysis, SPSS 16.0 statistical software (SPSS, Inc., Chicago, IL, USA) was used and the results are reported in the form of mean ±SD. The evaluations were repeated thrice while the differences between the groups were analyzed with the help of unpaired student’s t-test. The statistical significance was determined using unpaired student's t-tests and one-way ANOVA, with p < 0.05 indicating significance in all cases.

### Availability of data and material

All data are provided in this study and raw data can be requested from the corresponding author.

### Consent for publication

IEC, The Second Affiliated Hospital of Nanchang University, Nanchang, 330006, China approved the publication of data generated from this study.

## RESULTS

### The expression levels of HACE1 in patients with heart failure

**Figure 1:**
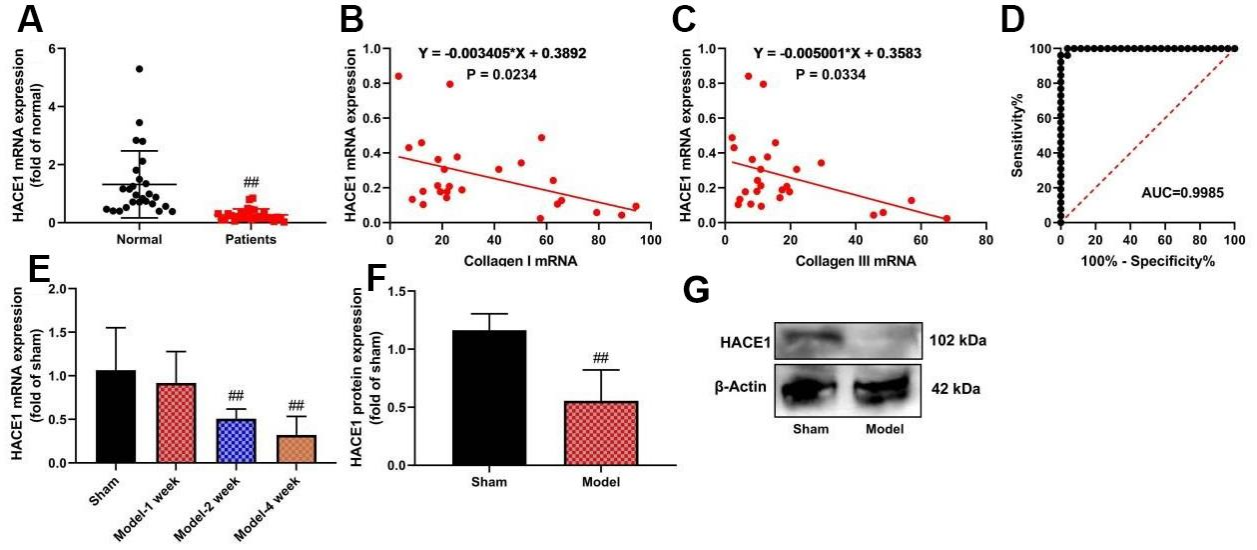
**The expression levels of HACE1 in patients with heart failure.** HACE1 mRNA expression (**A**), HACE1 related to collagen I or collagen III (**B**, **C**), ROC curve (**D**) in patients with Heart failure; HACE1 mRNA and promoted expression-rtPCR (**E**, **F**) and wester blot (**G**) in mice model of Heart failure. ##p<0.01 compared with normal group or sham group.

HACE1 mRNA expression was significantly inhibited in heart failure patients (p < 0.05) (Figure 1A). A negative correlation was observed between HACE1 serum expression levels and collagen I (p < 0.05) and collagen III (p < 0.05) in heart failure patients (Figure 1B, 1C). Receiver Operating Characteristic (ROC) curve analysis showed the diagnostic potential of HACE1 level (Figure 1D). HACE1 mRNA was up-regulated in mice with heart failure after 2-4 weeks of induction (p < 0.05) (Figure 1E). Expression of the HACE1 protein was detected in heart tissue samples from the heart failure mice model (Figure 1F).

### HACE1 reduced oxidative stress *in vitro* model

**Figure 2:**
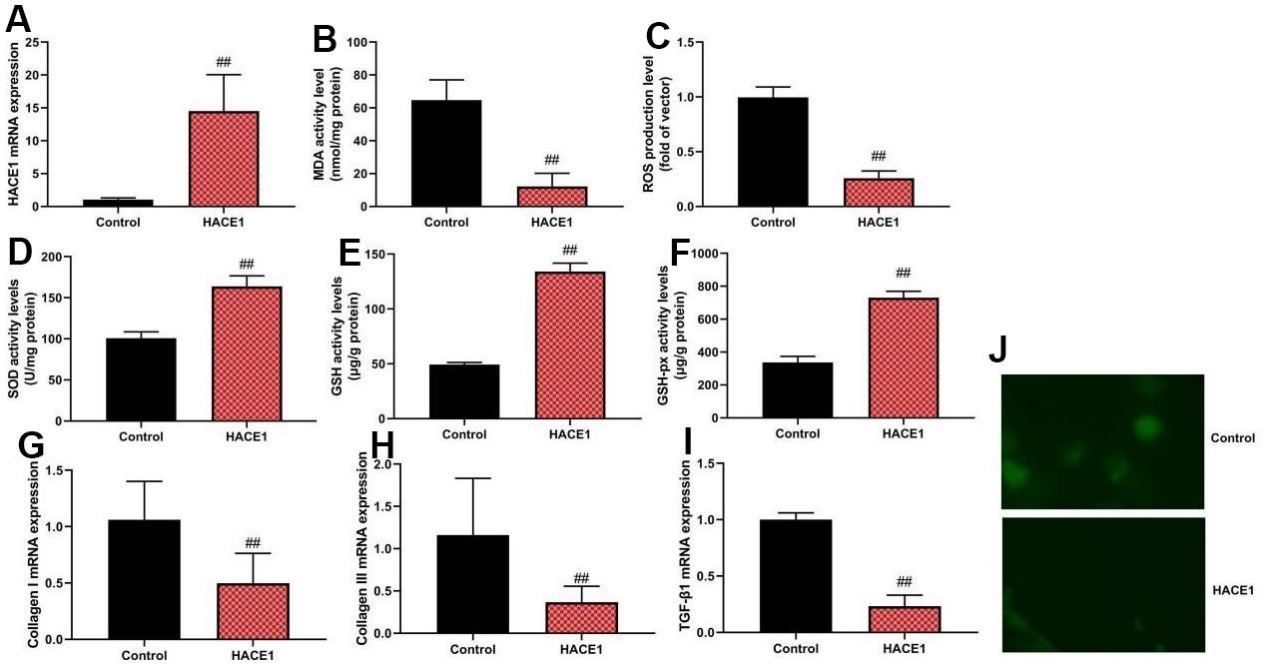
**HACE1 reduced oxidative stress *in vitro* model.** HACE1 mRNA expression (**A**), MDA, ROS, SOD, GSH and GSH-px levels (**B**–**F**), collagen I, collagen III and TGF-β mRNA expression (**G**–**I**). ##p<0.01 compared with control group. Immunofluorescence images of HACE1 (**J**).

Up-regulation of HACE1 via HACE1 plasmid increased ROS and MDA levels (p < 0.05) but reduced SOD, GSH, and GSH-PX levels (p < 0.05) in the *in vitro* heart failure model (Figure 2A–2F). HACE1 overexpression led to a decrease in collagen I, collagen III, and TGF-β mRNA expression (p < 0.05) (Figure 2G–2I).

### The inhibition of HACE1 promoted oxidative stress *in vitro* model

**Figure 3:**
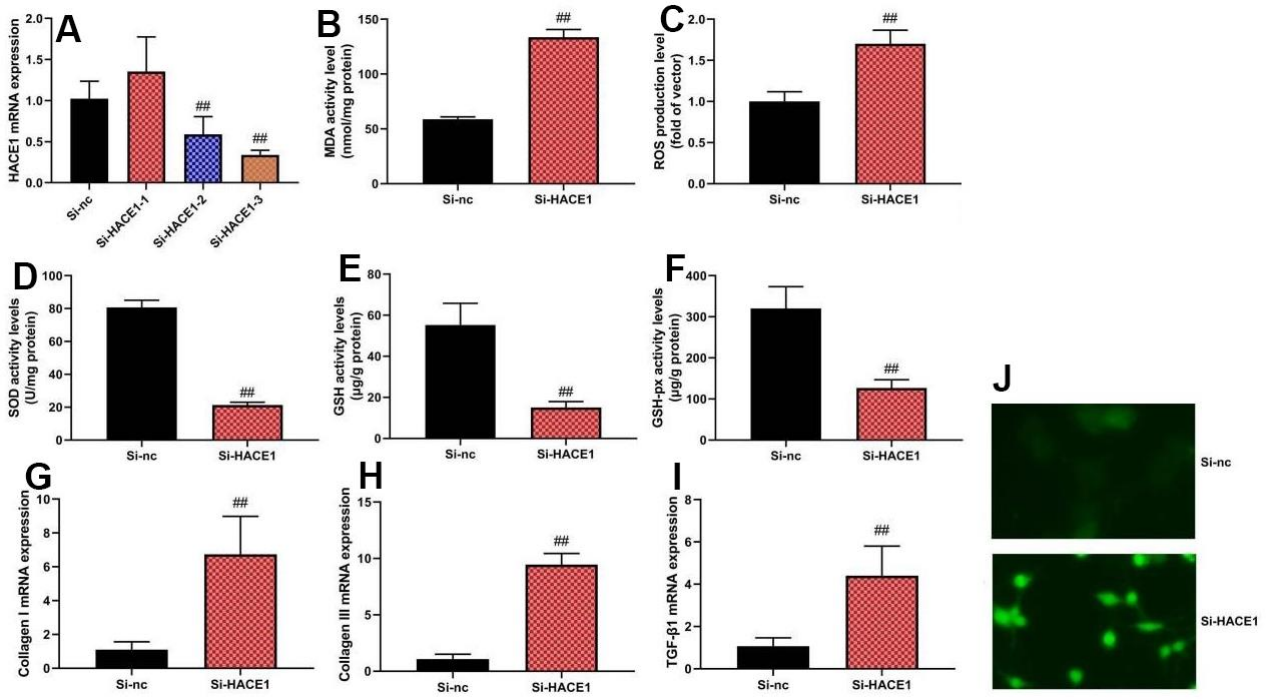
**The inhibition of HACE1 promoted oxidative stress *in vitro* model.** HACE1 mRNA expression (**A**), MDA, ROS, SOD, GSH and GSH-px levels (**B**–**F**), collagen I, collagen III and TGF-β mRNA expression (**G**–**I**). ##p<0.01 compared with si-nc group. Immunofluorescence images of si-HACE1 activity (**J**).

Knockdown of HACE1 via si-HACE1 decreased ROS and MDA levels (Figure 3A) (p < 0.05) but increased SOD, GSH, and GSH-PX levels (p < 0.05) in the *in vitro* heart failure model (Figure 3B–3F). HACE1 knockdown resulted in increased collagen I, collagen III, and TGF-β mRNA expression (p < 0.05) (Figure 3G–3I).

### HACE1 inhibited ferroptosis and mitochondrial damage *in vitro* model

**Figure 4:**
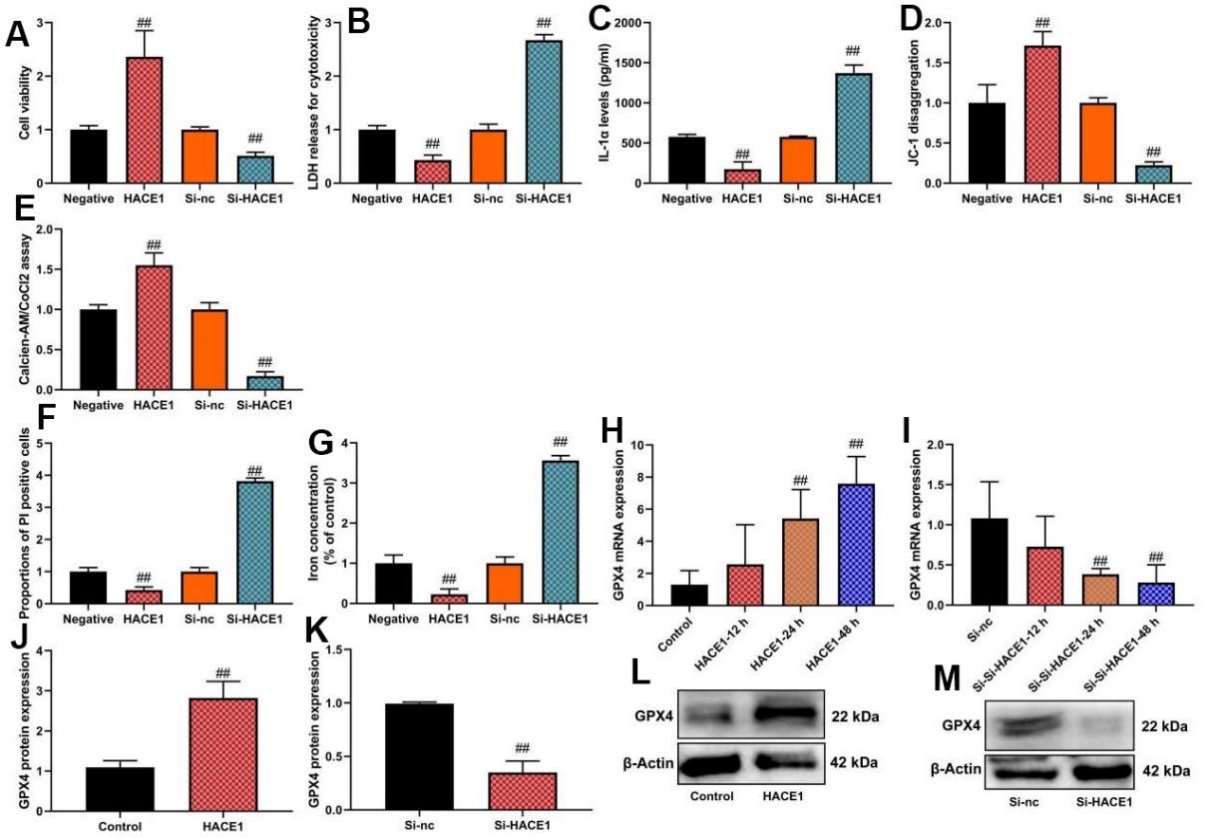
**HACE1 inhibited ferroptosis and mitochondrial damage *in vitro* model.** Cell viability (**A**), LDH activity (**B**), IL-1α level (**C**), JC-1 disaggregation (**D**), calcien-AM/CoCl2 rate level (**E**), PI positive cells (**F**), Iron concentration levels (**G**), GPX4 mRNA expression (**H**, **I**) and GPX4 protein expression (**J**, **K**) western blot images for GPX4 protein expression (**L**, **M**). ##p<0.01 compared with control group or si-nc group.

Up-regulation of HACE1 enhanced cell viability and promoted calcien-AM/CoCl2 levels (p < 0.05), reduced LDH activity and IL-1α levels (p < 0.05), and decreased PI-positive cells and iron concentration (p < 0.05) in the *in vitro* heart failure model (Figure 4A–4G). HACE1 overexpression increased GPX4 mRNA and protein expression (p < 0.05) (Figure 4H, 4J). HACE1 knockdown led to decreased cell viability, increased PI-positive cells and iron concentration (p < 0.05), and reduced GPX4 expression (p < 0.05) in the *in vitro* heart failure model (Figure 4A–4G, 4I, 4K).

### Sh-HACE1 could promote heart failure in mice model

**Figure 5:**
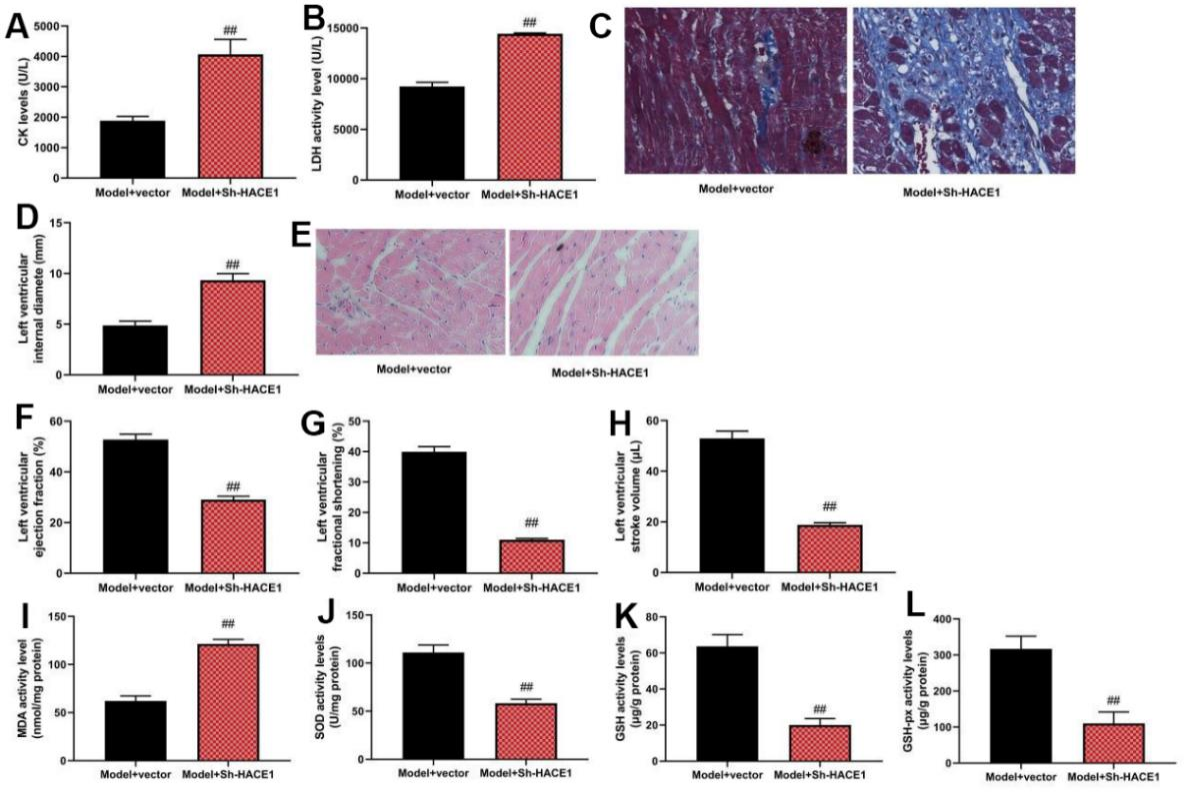
**Sh-HACE1 could promote heart failure in mice model.** CK and LDH activity levels (**A**, **B**), heat tissue injury (Masson and HE staining, **C**), left ventricular internal diameter (**D**), heat tissue injury (Masson and HE staining (**E**), left ventricular ejection fraction (**F**), left ventricular fractional shortening (**G**), left ventricular stroke volume (**H**), MDA/SOD/ GSH/GSH-PX levels in heat tissue (**I**–**L**) ##p<0.01 compared with model+vector group.

Sh-HACE1 aggravated CK and LDH activity (p < 0.05), increased left ventricular internal diameter, and caused heart tissue injury (p < 0.05). It also reduced ejection fraction, fractional shortening in left ventricles, and stroke volume of left ventricles in the heart failure mice model (p < 0.05). SOD, GSH, and GSH-PX levels were reduced by Sh-HACE1, while MDA levels were increased in heart tissue (p < 0.05) (Figure 5A–5L).

### NRF2 is one target for HACE1 in model of heart failure

**Figure 6:**
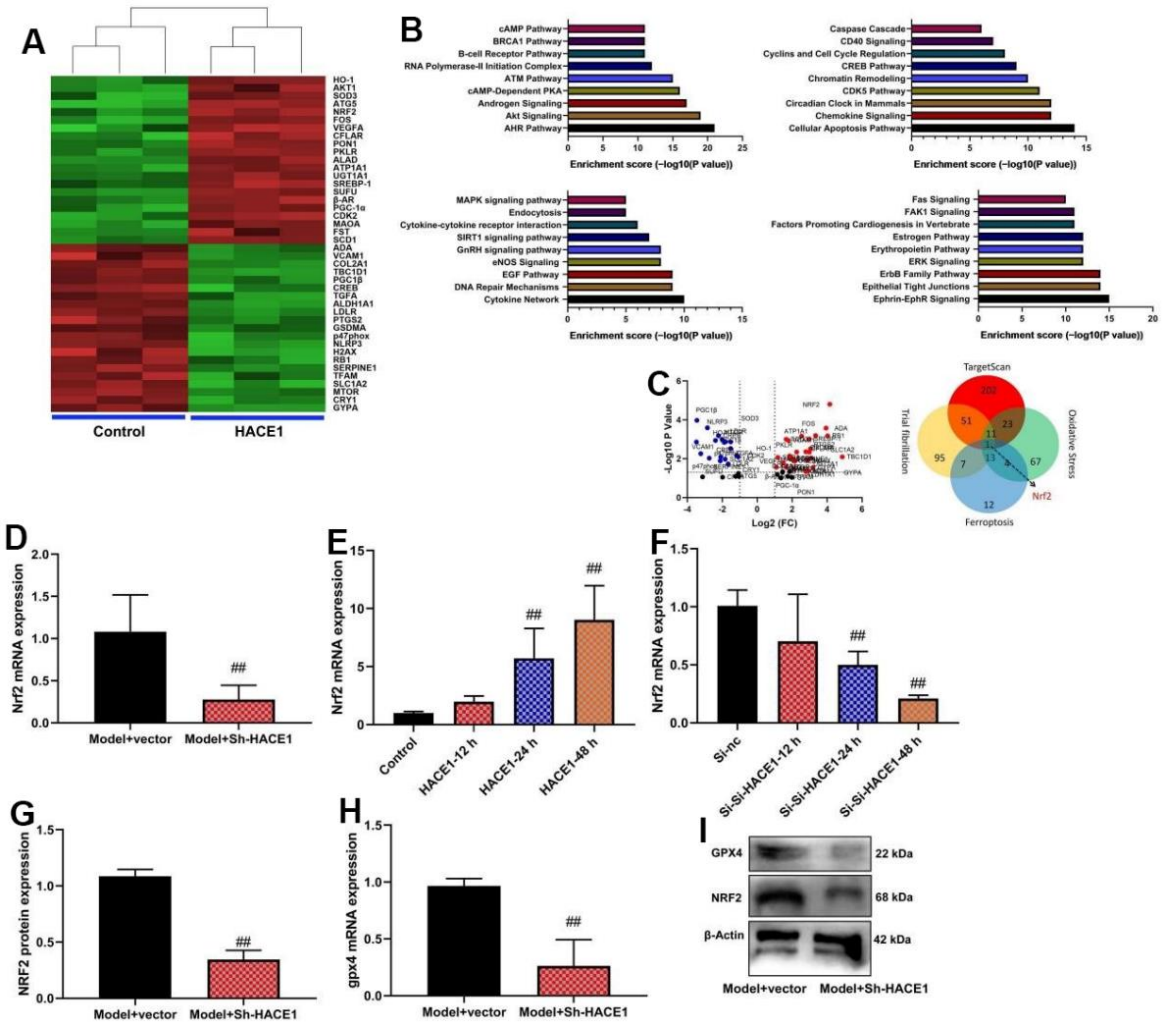
**NRF2 is one target for HACE1 in model of heart failure.** Microarray analysis (**A**–**C**), NRF2 mRNA expression (**D**–**F**), NRF2 and GPX4 protein expressions (**G**, **H**) GPX4 protein expressions in blot (**I**). ##p<0.01 compared with model+vector group or control group or si-nc group.

Sh-HACE1 reduced NRF2 mRNA expression in heart tissue (p < 0.05) (Figure 6D). HACE1 overexpression increased NRF2 mRNA expression (p < 0.05) (Figure 6E). HACE1 knockdown decreased NRF2 mRNA expression (p < 0.05) (Figure 6F). Sh-HACE1 reduced NRF2 and GPX4 protein expression in heart tissue (p < 0.05) (Figure 6H).

### HACE1 promoted NRF2 activity levels by its ubiquitination in model of heart failure

**Figure 7:**
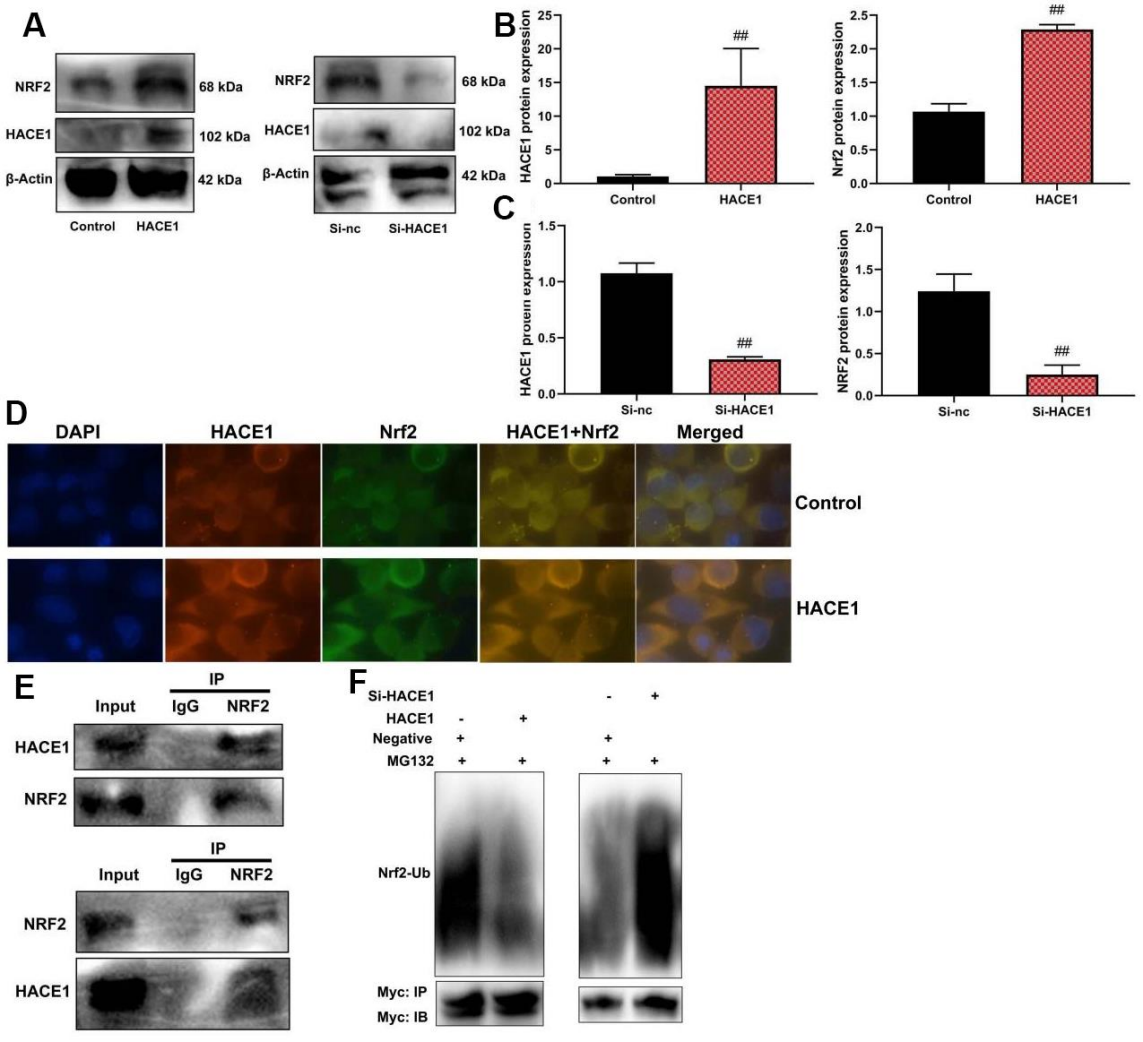
**HACE1 promoted NRF2 activity levels by its Ubiquitination in model of heart failure.** NRF2 protein expression (**A**–**C**), HACE1 and NRF2 expression (Microscopic display, (**D**), ACE1 protein connected with NRF2 protein (IP, **E**), NRF2 Ubiquitination (**F**). ##p<0.01 compared with control group or si-nc group.

HACE1 up-regulation led to increased expression of both HACE1 and NRF2 proteins in the *in vitro* model (p < 0.05) (Figure 7A–7C). Microscopic examination confirmed increased expression of HACE1 and NRF2 when HACE1 was up-regulated (p < 0.05) (Figure 7D). Immunoprecipitation (IP) showed a relationship between HACE1 and NRF2 proteins (Figure 7E). Up-regulation of HACE1 reduced the Ubiquitination of NRF2, while si-HACE1 promoted NRF2 Ubiquitination (p < 0.05) (Figure 7F).

### NRF2 inhibition and HACE1 impact

**Figure 8:**
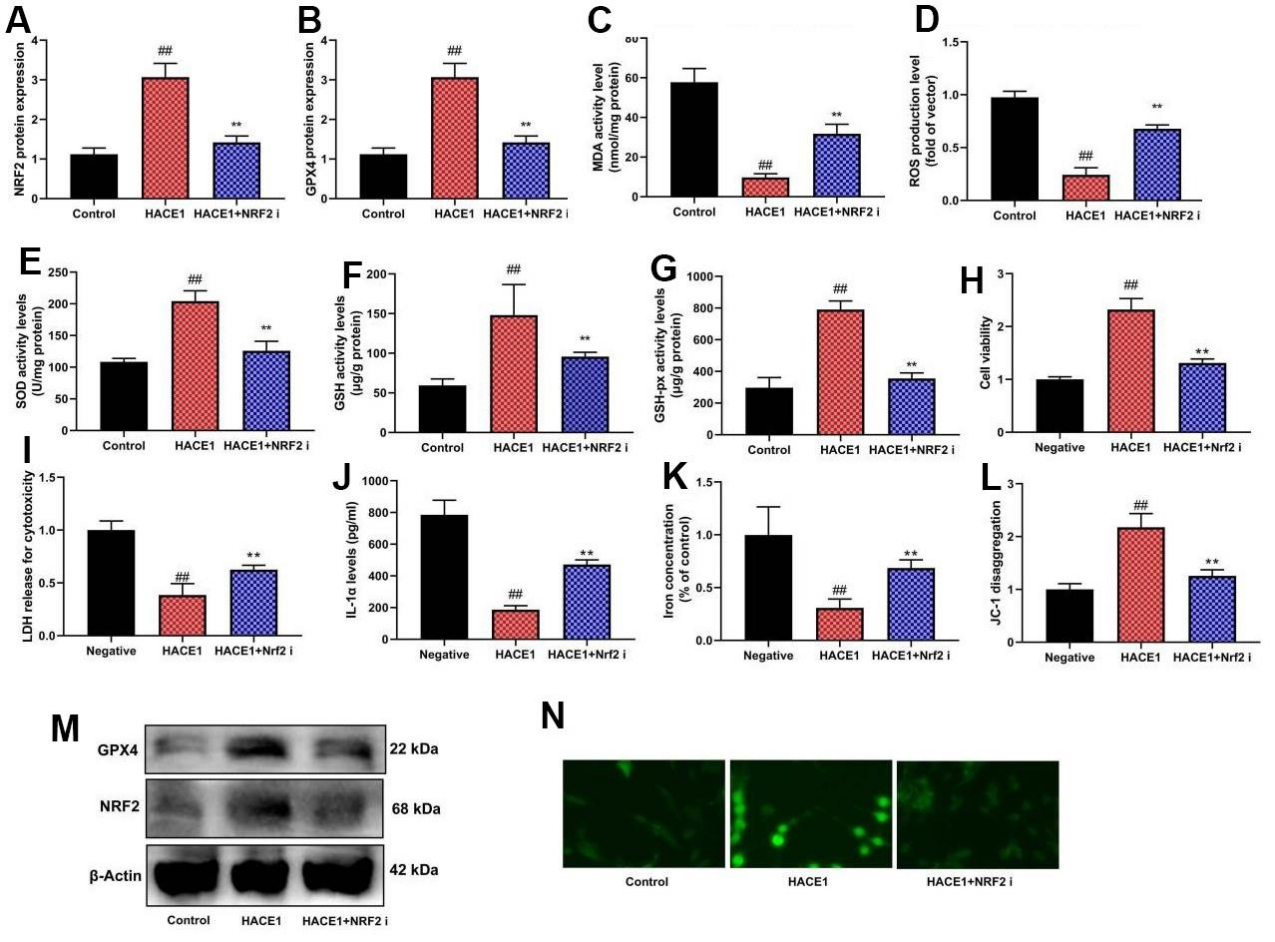
**The inhibition of NRF2 reduced the effects of HACE1 in model of heart failure.** NRF2 and GPX4 protein expression (**A**, **B**), MDA/ROS/SOD/GSH/GSH-PX levels (**C**–**G**), cell viability (**H**), LDH activity (**I**), IL-1α level (**J**), Iron concentration levels (**K**), JC-1 disaggregation (**L**). NRF2 and GPX4 protein expression in blot and immunofluorescence (**M**, **N**). ##p<0.01 compared with control group, **p<0.01 compared with HACE1 group.

**Figure 9:**
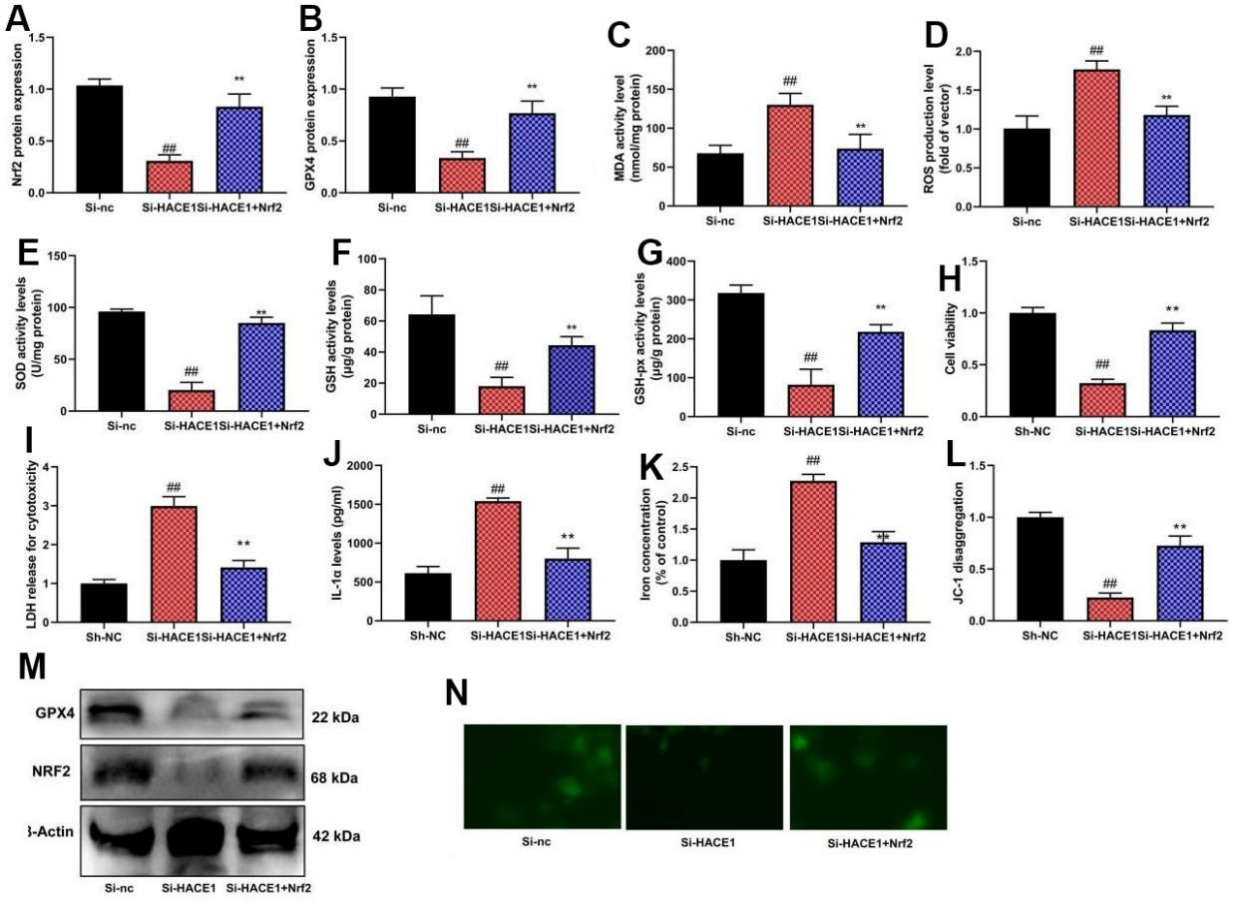
**The activation of NRF2 reduced the effects of si-HACE1 in model of heart failure.** NRF2 and GPX4 protein expression (**A**, **B**), MDA/ROS/SOD/GSH/GSH-PX levels (**C**–**G**), cell viability (**H**), LDH activity (**I**), IL-1α level (**J**), Iron concentration levels (**K**), JC-1 disaggregation (**L**). NRF2 and GPX4 protein expression in blot and immunofluorescence (**M**, **N**). ##p<0.01 compared with si-nc group, **p<0.01 compared with si-HACE1 group.

NRF2 inhibitor mitigated the impact of HACE1 on Nrf2 and GPX4 expression, oxidative stress, and ferroptosis *in vitro* (p < 0.05) (Figure 8). NRF2 agonist countered the impact of si-HACE1 on Nrf2 and GPX4 expression, oxidative stress, and ferroptosis *in vitro* (p < 0.05) (Figure 9).

## DISCUSSION

In clinical arrhythmias, heart failure is the most commonly observed on [14]. Due to aging population, heart failure has become common these days while its incidence rate and mortality levels are increasing on a remarkable number [15]. By 2010, 33.5 million patients were diagnosed with heart failure across the globe [16]. In the event of heart failure, the risks are high for stroke, cardiac arrest, thromboembolism etc., which in turn fatally impact the patients. These conditions further aggravate the economic burden of people and their society. Due to these reasons, the condition has gained attention in national and international levels in the field of CVDs [17]. As per the literature, atrial fibrosis, oxidative stress, cardiac remodeling, ion channel abnormalities and other such pathological changes occur due to multiple diseases including CHD, diabetes, heart failure, hypertension and so on. Further, it also results in the blood’s hypercoagulability which eventually leads to heart failure [17, 18]. In current study, an inhibitory effect was expressed by HACE1 mRNA in heart failure model. In *in-vitro* studies, the oxidative stress got reduced by the expression of HACE1. However, the Sh-HACE1 tend to aggravate the heart failure problem in mice model. According to Razaghi et al., the cardiac development in zebrafish was regulated by HACE1 via ROS-oxidative stress [19]. As per these findings, the HACE1 was found to be one of the crucial regulatory genes that mitigate the oxidative stress in case of heart failure model.

With remarkable differences from autophagy and apoptosis, Ferroptosis is a novel type of programmed cell death process that was found recently [20]. The lipid metabolism pathway of this novel cell death process primarily manifests in the increased levels of intracellular biological markers such as such as ROS, ACSl4 and lpcat3 [21]. GPx-4 is a type of peroxidase reductase that contains selenium and has the potential to degrade the lipid peroxides into hydroxyl lipids that are non-toxic in nature. Being a core-level protease enzyme, it tends to antagonize the iron death [21]. When the activity of GPx-4 is inhibited, it enhances the lipid peroxide levels and eventually, the activation of Ferroptosis occurs [22]. The current study results infer that both ferroptosis and mitochondrial damage were inhibited by the expression of HACE1 in *in-vitro* model. Ugarteburu et al. found the regulation of HACE1 in mitophagy and oxidative stress [23]. So, it is inferred that the inhibition of ferroptosis and mitochondrial damage was accomplished by HACE1 by preventing the ROS-oxidative stress in heart failure model.

Being an endogenous cell resistance regulator against oxidants, NRF2 also functions as a crucial transcription factor [24]. When NRF2 is activated, it results in the gene-level expression of antioxidant defense enzyme HO-1 [25] and other such antioxidant response elements (ARE). In literature, it has been concluded that NRF2 gets inhibited as a result of heavy oxidative stress, fibrosis and hypertrophy. So, it is well understood that NRF2 enacts a critical role in atomic fibrosis pathway [26, 27]. Through current study results, NRF2 was identified as a target for HACE1 in heart failure model. By ubiquitination, the HACE1 boosted the activity levels of NRF2 in heart failure model. According to Da et al., NRF2 gets activated by HACE1 in glioma cells [28]. The current study outcomes confirm the promotion of NRF2’s activities by HACE1, while the latter inhibited the ubiquitination of the former in order to mitigate the mitochondrial damage and ferroptosis by inactivating the ROS-oxidative stress in heart failure model.

Limitations of this study include its heavy reliance on animal models, necessitating further clinical validation to establish the practical utility of HACE1 in diagnosing and prognosing heart failure in humans. The intricate mechanisms underpinning heart failure remain incompletely understood, emphasizing the need for more in-depth research. Additionally, the generalizability of HACE1's role to all heart diseases may not hold true given the heterogeneity of these conditions. In the future, extensive clinical studies, mechanistic elucidation, humanized models, and investigations into therapeutic potential should guide research efforts towards a better understanding and management of heart diseases.

Among various clinical arrhythmias, heart failure is associated with increased risks of circulatory embolism and ischemic stroke. This study aimed to investigate the role of HACE1 in heart failure and its potential therapeutic applications. The study compared the expression levels of HACE1 between heart failure patients (experimental group) and normal individuals (normal group). The results revealed a significant down-regulation of HACE1 in the heart failure patients, suggesting its possible involvement in the disease. In *in-vitro* experiments, the overexpression of HACE1 mitigated oxidative stress induced by ROS, while down-regulation of HACE1 increased oxidative stress. Additionally, HACE1 triggered the NRF2 pathway, promoting the expression of GPX4 protein, which further mitigated oxidative stress and ferroptosis. Furthermore, *in-vivo* experimentation on mice showed that down-regulation of HACE1 using shRNA aggravated heart failure and enhanced oxidative stress. Conversely, up-regulation of HACE1 inhibited ferroptosis and mitochondrial damage.

## References

[1] Di Monaco A, Vitulano N, Troisi F, Quadrini F, Guida P, Grimaldi M. Long-term mortality of patients ablated for atrial fibrillation: a retrospective, population-based epidemiological study in Apulia, Italy. BMJ Open. 2022; 12:e058325. 10.1136/bmjopen-2021-058325 35393325 PMC8991055

[2] Galvain T, Hill R, Donegan S, Lisboa P, Lip GYH, Czanner G. The management of anticoagulants in patients with atrial fibrillation and history of falls or risk of falls: protocol for a systematic review and meta-analysis. Syst Rev. 2022; 11:63. 10.1186/s13643-022-01937-0 35395931 PMC8991693

[3] López-Gálvez R, Rivera-Caravaca JM, Anguita Sánchez M, Sanmartín Fernández M, Rafols C, Pérez-Cabeza AI, Barón Esquivias G, Lekuona Goya I, Vázquez Rodríguez JM, Cosín Sales J, Arribas Ynsaurriaga F, Barrios V, Freixa-Pamias R, Marín F. Use of rivaroxaban attenuates renal function impairment in patients with atrial fibrillation: insights of the EMIR study. Eur J Clin Invest. 2022; 52:e13788. 10.1111/eci.13788 35395094

[4] Wu S, Huang N, Chen X, Jiang S, Zhang W, Hu W, Su J, Dai H, Gu P, Huang X, Du X, Li R, Zheng Q, et al. Association between Body Mass Index and Clinical Outcomes in Patients with Non-valvular Atrial Fibrillation Receiving Direct Oral Anticoagulants: A New Piece of Evidence on the Obesity Paradox from China. Cardiovasc Drugs Ther. 2023; 37:715–27. 10.1007/s10557-022-07332-0 35394582

[5] Zhong X, Jiao H, Zhao D, Teng J, Yang M. A Retrospective Study to Determine the Association Between Serum Albumin Levels and Atrial Fibrillation by Sex in 950 Patients from a Single Center in China. Med Sci Monit. 2022; 28:e935347. 10.12659/MSM.935347 35393388 PMC9004327

[6] Lee S, Choi E, Cha MJ, Hwang KC. Looking into a conceptual framework of ROS-miRNA-atrial fibrillation. Int J Mol Sci. 2014; 15:21754–76. 10.3390/ijms151221754 25431922 PMC4284676

[7] Sovari AA, Morita N, Karagueuzian HS. Apocynin: a potent NADPH oxidase inhibitor for the management of atrial fibrillation. Redox Rep. 2008; 13:242–5. 10.1179/135100008X309000 19017463

[8] Yoo S, Aistrup G, Shiferaw Y, Ng J, Mohler PJ, Hund TJ, Waugh T, Browne S, Gussak G, Gilani M, Knight BP, Passman R, Goldberger JJ, et al. Oxidative stress creates a unique, CaMKII-mediated substrate for atrial fibrillation in heart failure. JCI Insight. 2018; 3:e120728. 10.1172/jci.insight.120728 30385719 PMC6238754

[9] Deng HX. HACE1, RAC1, and what else in the pathogenesis of SPPRS? Neurol Genet. 2019; 5:e326. 10.1212/NXG.0000000000000326 31321299 PMC6561752

[10] El-Naggar AM, Clarkson PW, Negri GL, Turgu B, Zhang F, Anglesio MS, Sorensen PH. HACE1 is a potential tumor suppressor in osteosarcoma. Cell Death Dis. 2019; 10:21. 10.1038/s41419-018-1276-4 30622235 PMC6325116

[11] Turgu B, Zhang F, El-Naggar A, Negri GL, Kogler M, Tortola L, Johnson F, Ng T, Li A, Yapp D, Lockwood W, Martinez D, Maris JM, et al. HACE1 blocks HIF1α accumulation under hypoxia in a RAC1 dependent manner. Oncogene. 2021; 40:1988–2001. 10.1038/s41388-021-01680-1 33603169 PMC7979542

[12] Kogler M, Tortola L, Negri GL, Leopoldi A, El-Naggar AM, Mereiter S, Gomez-Diaz C, Nitsch R, Tortora D, Kavirayani AM, Gapp BV, Rao S, Uribesalgo I, et al. HACE1 Prevents Lung Carcinogenesis via Inhibition of RAC-Family GTPases. Cancer Res. 2020; 80:3009–22. 10.1158/0008-5472.CAN-19-2270 32366477 PMC7611202

[13] Pan JA, Lin H, Yu JY, Zhang HL, Zhang JF, Wang CQ, Gu J. MiR-21-3p Inhibits Adipose Browning by Targeting FGFR1 and Aggravates Atrial Fibrosis in Diabetes. Oxid Med Cell Longev. 2021; 2021:9987219. 10.1155/2021/9987219 34484568 PMC8413063

[14] Ly OT, Chen H, Brown GE, Hong L, Wang X, Han YD, Pavel MA, Sridhar A, Maienschein-Cline M, Chalazan B, Ong SG, Abdelhady K, Massad M, et al. Mutant ANP induces mitochondrial and ion channel remodeling in a human iPSC-derived atrial fibrillation model. JCI Insight. 2022; 7:e155640. 10.1172/jci.insight.155640 35393944 PMC9057627

[15] Schweizer J, Arnold M, König IR, Bicvic A, Westphal LP, Schütz V, Inauen C, Scherrer N, Luft A, Galovic M, Ferreira Atuesta C, Pokorny T, Arnold M, et al. Measurement of Midregional Pro-Atrial Natriuretic Peptide to Discover Atrial Fibrillation in Patients With Ischemic Stroke. J Am Coll Cardiol. 2022; 79:1369–81. 10.1016/j.jacc.2022.01.042 35393018

[16] Vaitsiakhovich T, Coleman CI, Kleinjung F, Vardar B, Schaefer B. Worsening of kidney function in patients with atrial fibrillation and chronic kidney disease: evidence from the real-world CALLIPER study. Curr Med Res Opin. 2022; 38:937–45. 10.1080/03007995.2022.2061705 35392744

[17] Liang X, Zhang Q, Wang X, Yuan M, Zhang Y, Xu Z, Li G, Liu T. Reactive oxygen species mediated oxidative stress links diabetes and atrial fibrillation. Mol Med Rep. 2018; 17:4933–40. 10.3892/mmr.2018.8472 29393403 PMC5865952

[18] Lu G, Xu C, Tang K, Zhang J, Li Q, Peng L, Wang Y, Huang Z, Gao X. H2S inhibits angiotensin II-induced atrial Kv1.5 upregulation by attenuating Nox4-mediated ROS generation during atrial fibrillation. Biochem Biophys Res Commun. 2017; 483:534–40. 10.1016/j.bbrc.2016.12.110 28011270

[19] Razaghi B, Steele SL, Prykhozhij SV, Stoyek MR, Hill JA, Cooper MD, McDonald L, Lin W, Daugaard M, Crapoulet N, Chacko S, Lewis SM, Scott IC, et al. hace1 Influences zebrafish cardiac development via ROS-dependent mechanisms. Dev Dyn. 2018; 247:289–303. 10.1002/dvdy.24600 29024245

[20] Dai C, Kong B, Qin T, Xiao Z, Fang J, Gong Y, Zhu J, Liu Q, Fu H, Meng H, Shuai W, Huang H. Inhibition of ferroptosis reduces susceptibility to frequent excessive alcohol consumption-induced atrial fibrillation. Toxicology. 2022; 465:153055. 10.1016/j.tox.2021.153055 34864093

[21] Fang J, Kong B, Shuai W, Xiao Z, Dai C, Qin T, Gong Y, Zhu J, Liu Q, Huang H. Ferroportin-mediated ferroptosis involved in new-onset atrial fibrillation with LPS-induced endotoxemia. Eur J Pharmacol. 2021; 913:174622. 10.1016/j.ejphar.2021.174622 34748769

[22] Zhang L, Guo Y, Xiaokereti J, Cao G, Li H, Sun H, Li K, Zhou X, Tang B. Ganglionated Plexi Ablation Suppresses Chronic Obstructive Sleep Apnea-Related Atrial Fibrillation by Inhibiting Cardiac Autonomic Hyperactivation. Front Physiol. 2021; 12:640295. 10.3389/fphys.2021.640295 33897452 PMC8063039

[23] Ugarteburu O, Sánchez-Vilés M, Ramos J, Barcos-Rodríguez T, Garrabou G, García-Villoria J, Ribes A, Tort F. Physiopathological Bases of the Disease Caused by HACE1 Mutations: Alterations in Autophagy, Mitophagy and Oxidative Stress Response. J Clin Med. 2020; 9:913. 10.3390/jcm9040913 32225089 PMC7231286

[24] Chen M, Zhong J, Wang Z, Xu H, Chen H, Sun X, Lu Y, Chen L, Xie X, Zheng L. Fibroblast Growth Factor 21 Protects Against Atrial Remodeling via Reducing Oxidative Stress. Front Cardiovasc Med. 2021; 8:720581. 10.3389/fcvm.2021.720581 34708083 PMC8542911

[25] Qiu H, Wu H, Ma J, Cao H, Huang L, Qiu W, Peng Y, Ding C. DL-3-n-Butylphthalide reduces atrial fibrillation susceptibility by inhibiting atrial structural remodeling in rats with heart failure. Naunyn Schmiedebergs Arch Pharmacol. 2018; 391:323–34. 10.1007/s00210-017-1457-1 29290021

[26] Rochette L, Lorin J, Zeller M, Guilland JC, Lorgis L, Cottin Y, Vergely C. Nitric oxide synthase inhibition and oxidative stress in cardiovascular diseases: possible therapeutic targets? Pharmacol Ther. 2013; 140:239–57. 10.1016/j.pharmthera.2013.07.004 23859953

[27] Xu L, Fan Y, Wu L, Zhang C, Chu M, Wang Y, Zhuang W. Exosomes from Bone Marrow Mesenchymal Stem Cells with Overexpressed Nrf2 Inhibit Cardiac Fibrosis in Rats with Atrial Fibrillation. Cardiovasc Ther. 2022; 2022:2687807. 10.1155/2022/2687807 35360547 PMC8941574

[28] Da C, Pu J, Liu Z, Wei J, Qu Y, Wu Y, Shi B, Yang J, He N, Hou P. HACE1-mediated NRF2 activation causes enhanced malignant phenotypes and decreased radiosensitivity of glioma cells. Signal Transduct Target Ther. 2021; 6:399. 10.1038/s41392-021-00793-z 34815381 PMC8611003

